# Supplementary material for: Button shear testing for adhesion measurements of 2D materials
Source: Nat Commun. 2024 Mar 18;15:2430. doi: 10.1038/s41467-024-46136-8 (PMC10948857; doi:10.1038/s41467-024-46136-8)
Supplement: Supplementary file 1 — Supplementary Information [file 41467_2024_46136_MOESM1_ESM.pdf]

Supplementary Information

Button Shear Testing for Adhesion Measurements of 2D Materials

Josef Schätz[1,2], Navin Nayi[1], Jonas Weber[3,4], Christoph Metzke[3,5], Sebastian Lukas[2], Jürgen Walter[1], Tim Schaffus[1], Fabian Streb[1], Annika Grundmann[6], Holger Kalisch[6], Michael Heuken[6,7], Andrei Vescan[6], Stephan Pindl[1], and Max C. Lemme[2,8]*

[1]Infineon Technologies AG, Wernerwerkstraße 2, 93049 Regensburg, Germany

[2]Chair of Electronic Devices, RWTH Aachen University, Otto-Blumenthal-Str. 25, 52074 Aachen, Germany

[3]Department of Electrical Engineering and Media Technology, Deggendorf Institute of Technology, Dieter-Görlitz-Platz 1, 94469 Deggendorf, Germany

[4]Department of Applied Physics, University of Barcelona, Martí i Franquès 1, 08028 Barcelona, Spain

[5]Department of Electrical Engineering, Helmut Schmidt University/University of the Federal Armed Forces Hamburg, Holstenhofweg 85, 22043 Hamburg, Germany

[6]Compound Semiconductor Technology, RWTH Aachen University, Sommerfeldstr. 18, 52074 Aachen, Germany

[7]AIXTRON SE, Dornkaulstr. 2, 52134 Herzogenrath, Germany

[8]AMO GmbH, Advanced Microelectronic Center Aachen, Otto-Blumenthal-Str. 25, 52074 Aachen, Germany

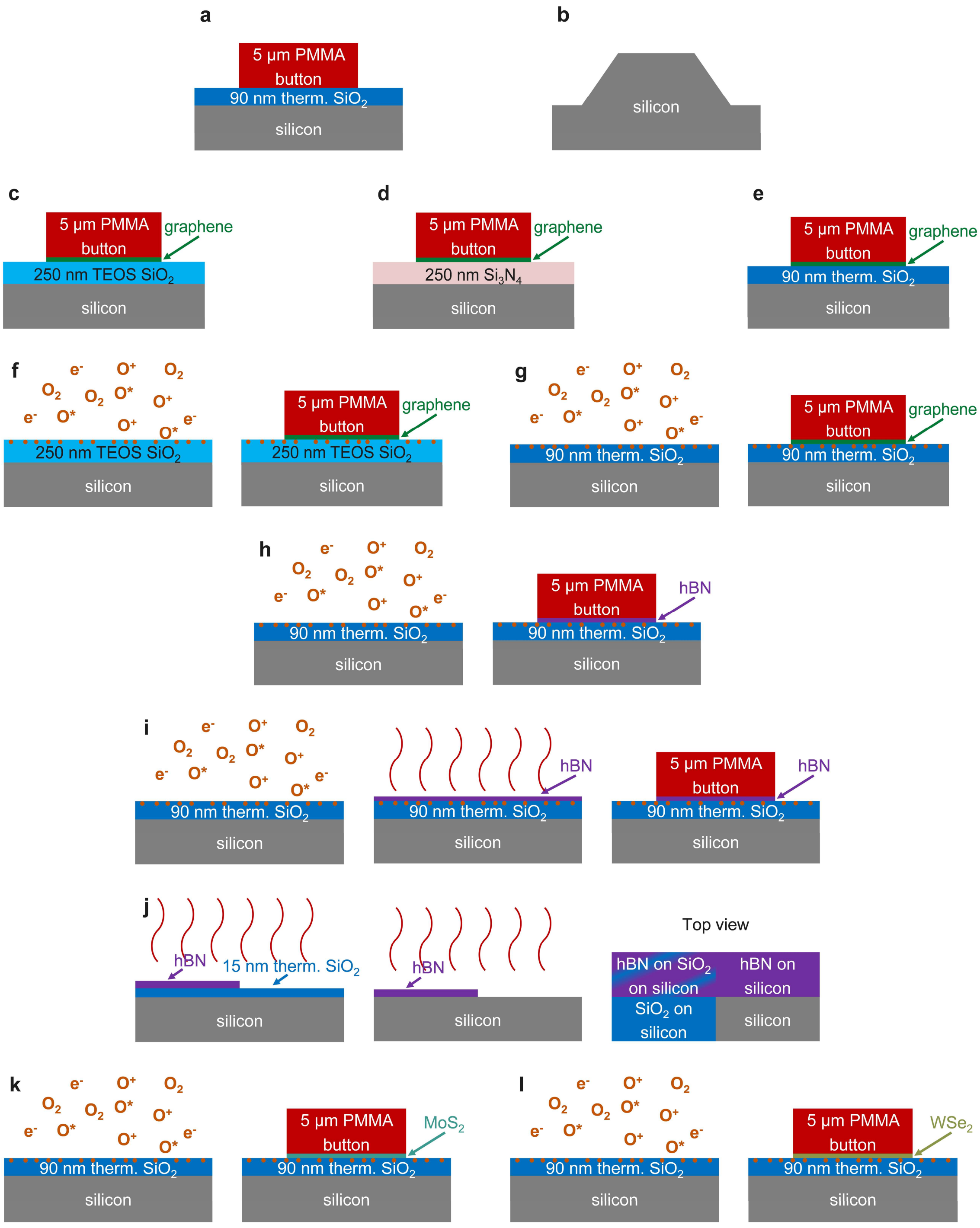

Supplementary Fig. 1 Schematic cross sections of all samples.

Schematic cross sections of **a** reference samples with PMMA button on 90 nm thermal SiO_2 to define button dimensions and shear speed and **b** calibration samples with a step in silicon to define the button shear tester cartridge. Samples with graphene on **c** TEOS SiO_2, **d** Si_3N_4, and **e** thermal SiO_2. Samples with graphene and O2 plasma as pretreatment on **f** TEOS SiO_2 and **g** thermal SiO_2. Sample with hBN on thermal SiO_2 with O2 plasma as pretreatment and **h** without and **i** with thermal anneal after hBN transfer. **j** Samples for SThM measurement with hBN on 15 nm thermal SiO_2. Sample with **k** MoS_2 and **l** WSe_2 on thermal SiO_2 with O2 plasma as pretreatment.

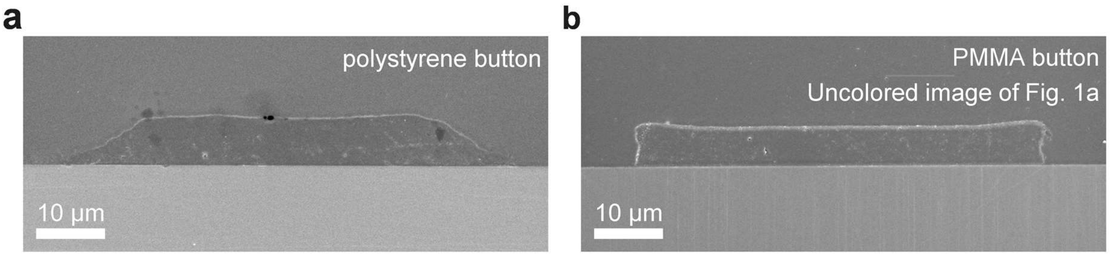

Supplementary Fig. 2 Button cross-sections.

a Scanning electron microscopy (SEM) cross-section of a polystyrene button. The pronounced trapezoidal cross-section is undesired for shear head contacting. **b** SEM cross-section of a polymethyl methacrylate (PMMA) button. The cuboid cross-section enables a reliable shear head contacting and button shear testing.

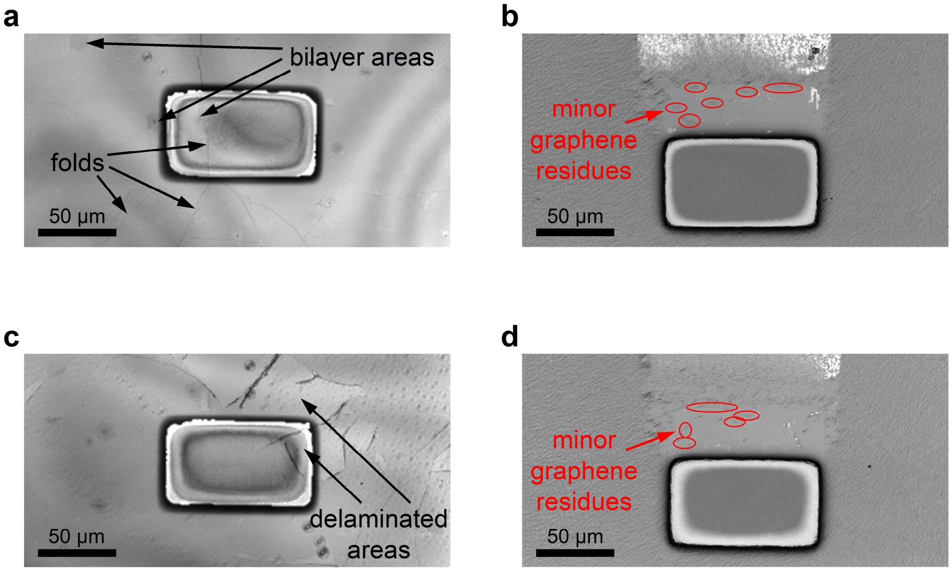

Supplementary Fig. 3 Assessment of buttons before and after button shear testing.

Exemplary laser scanning microscope images of buttons before graphene structuring (**a**, **c**) and after button shear testing of the same buttons (**b**, **d**). A button like in **a** is included in the assessment of shear strength τ_C because we accepted some folds and bilayer areas. We excluded buttons like in **c** because cracks and delaminated areas underneath the button before testing were not accepted. For all buttons the delamination occurs at the substrate - 2D material interface which can be seen in the images after button shear testing (**b**, **d**). Only minor areas with 2D material are left on the surface (highlighted in red in **b** and **d**).

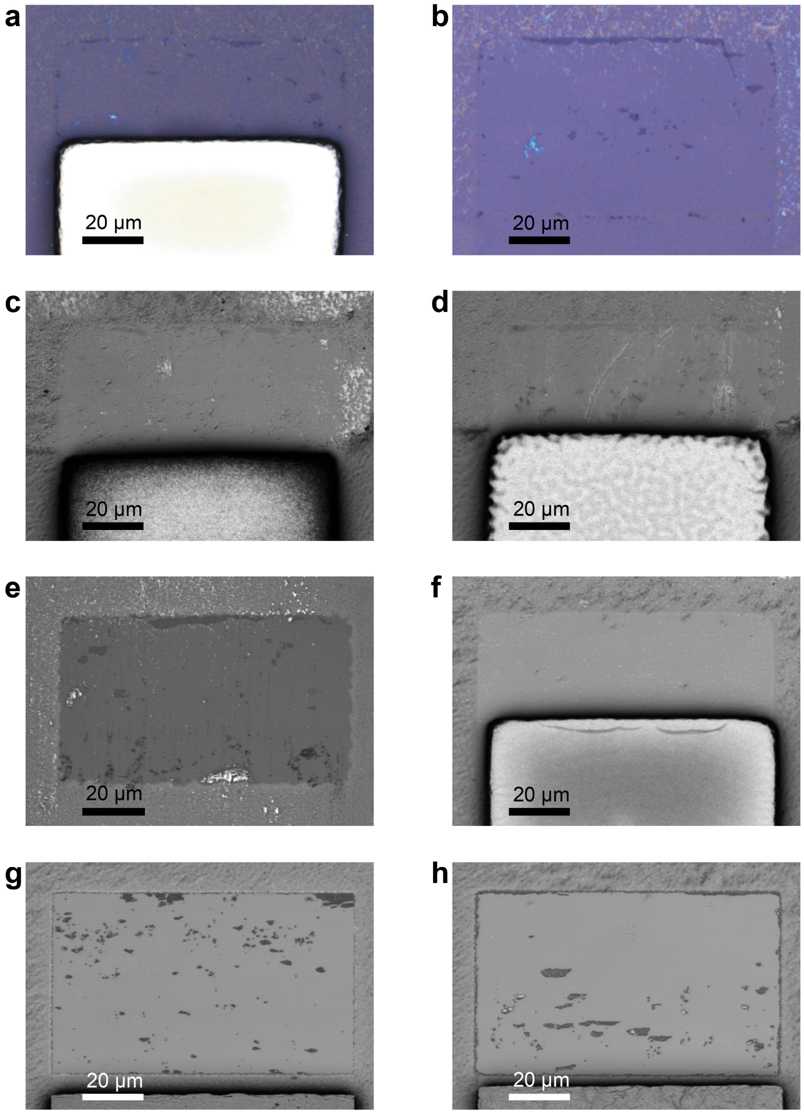

Supplementary Fig. 4 Microscope images of sheared interfaces after button shear testing.

a Optical microscope image of graphene on thermal SiO_2 without O_2 plasma treatment and **b** graphene on thermal SiO_2 with O_2 plasma treatment. Graphene is sheared from the thermal SiO_2 substrate at the major part of the button area. Minor areas with graphene remain on the surface and are visible as dark blue areas, especially on the contacted button edge (top edge in the images). Laser scanning microscope images of **c** graphene on TEOS SiO_2 without O_2 plasma treatment, **d** graphene on TEOS SiO_2 with O_2 plasma treatment, **e** graphene on Si_3N_4, **f** hBN on thermal SiO_2 with O_2 plasma treatment, **g** MoS_2 on thermal SiO_2 with O_2 plasma treatment, and **h** WSe_2 on thermal SiO_2 with O_2 plasma treatment. On all samples, only minor 2D material residues are detected. Delamination occurred predominantly at the substrate - 2D material interface.

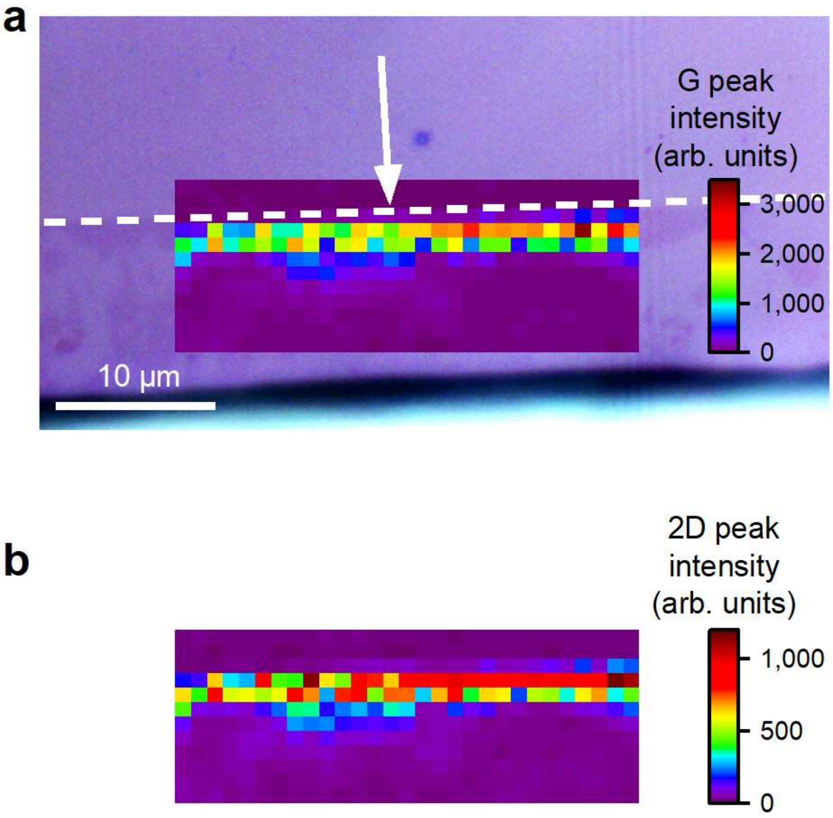

Supplementary Fig. 5 Raman measurement of sheared interface graphene - thermal SiO_2.

a Overlap of optical microscope image and G peak intensity heat map. The button edge position before shear testing is indicated as a white dashed line and the shear direction of the shear head as a white arrow. **b** 2D peak intensity of the same area. Graphene residues remain at the contacted edge and nearly no graphene is detected at a few µm distance from the contacted edge.

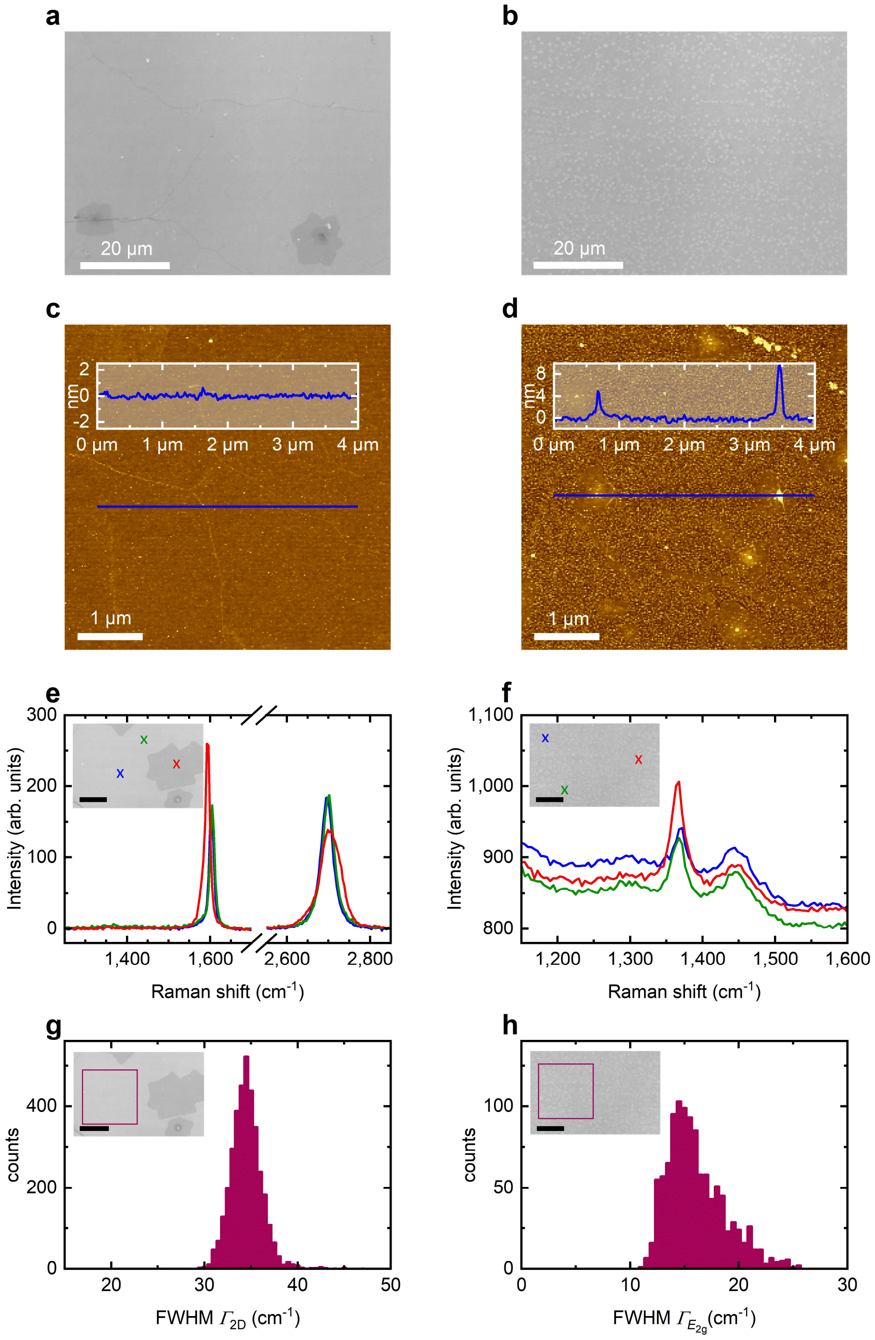

Supplementary Fig. 6 Investigation of graphene and hBN on thermal SiO_2.

Laser scanning microscope images of **a** graphene and **b** hBN on thermal SiO_2. The graphene in this work consists of predominantly monolayer graphene with a few bi- and multi-layer areas with a lateral size of approx. 10 µm and some folds. The hBN in this work consists of monolayer hBN with a high density of multilayer areas with a lateral size of approx. 1 µm. Atomic force microscopy (AFM) measurements of **c** graphene and **d** hBN. Some additional minor folds on the graphene monolayer area become visible. The hBN multilayer areas are detected with a height of up to 10 nm thickness. Exemplary Raman measurements of **e** graphene and **f** hBN. No D peak was detected on graphene and an I_{2D}/I_G ratio of 1.08 on the monolayer area and 0.53 on the bilayer area is found. Measurements on three arbitrary points on hBN reveal a small E_{2g} peak with varying intensity. Statistical distribution of the full-width at half-maximum (FWHM) Γ of **g** 2D peaks of graphene and **h** E_{2g} peaks of hBN. Insets in **e**-**h** indicate Raman measurement position with scale bar 10 µm.

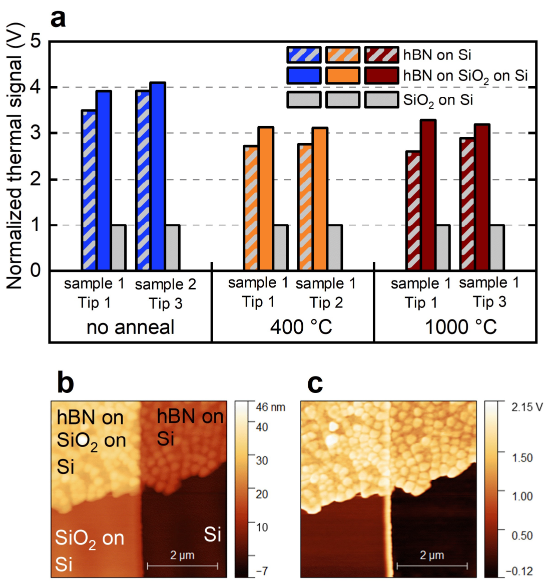

Supplementary Fig. 7 Scanning thermal microscopy (SThM) measurements of hBN on SiO_2 on Si and of hBN on Si.

a Thermal signal of SThM measurements on different stacks before anneal (dark blue), after 400 °C (orange), and after 1000 °C (dark red) anneal. The signal is normalized to the signal of SiO_2 on Si. The thermal resistance is reduced after a 400 °C anneal, both for a multilayer hBN on SiO_2 on Si stack and a multilayer hBN on Si stack, and remains unchanged after an additional anneal at 1000 °C. Two samples (sample 1 and sample 2) with same sample fabrication process were measured before anneal to check the reproducibility of the measurement. Also, different tips (Tip 1, Tip 2, and Tip 3) were used to exclude an influence of the tip. **b** Exemplary topography map and **c** thermal signal map for the different stacks. These maps were collected on sample 1 after a 400 °C anneal.

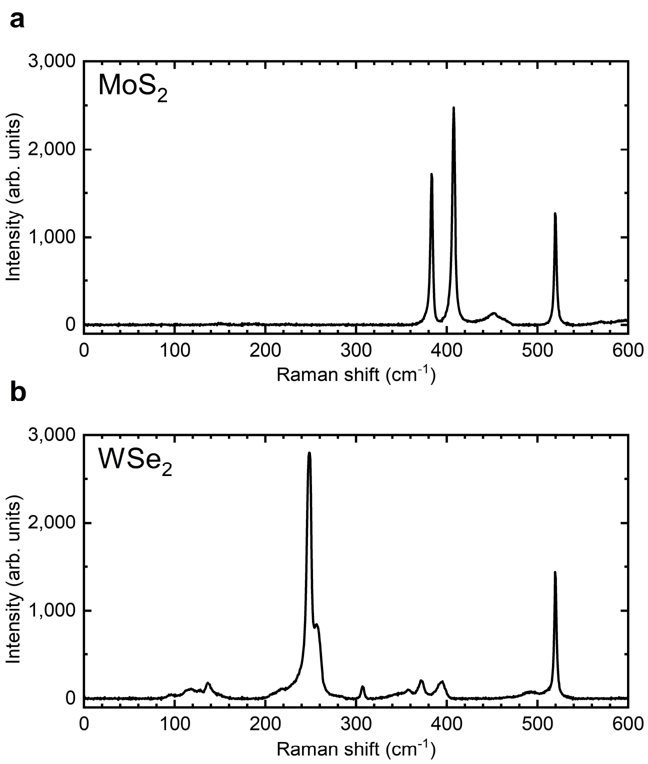

Supplementary Fig. 8 Raman spectra of transition metal dichalcogenides (TMDC).

Raman spectra of **a** MoS_2 and **b** WSe_2 on therm. SiO_2 on Si.
